# Systematic analysis of lncRNA–miRNA–mRNA competing endogenous RNA network identifies four-lncRNA signature as a prognostic biomarker for breast cancer

**DOI:** 10.1186/s12967-018-1640-2

**Published:** 2018-09-27

**Authors:** Chun-Ni Fan, Lei Ma, Ning Liu

**Affiliations:** Department of Breast Surgery, China-Japan Union Hospital of Jilin University, NO. 126, Xian Tai Street, Changchun, 130033 Jilin China

**Keywords:** Breast cancer, Long non-coding RNA, Prognosis, Competing endogenous RNA network, Overall survival

## Abstract

**Background:**

Increasing evidence has underscored the role of long non-coding RNAs (lncRNAs) acting as competing endogenous RNAs (ceRNAs) in the development and progression of tumors. Nevertheless, lncRNA biomarkers in lncRNA-related ceRNA network that can predict the prognosis of breast cancer (BC) are still lacking. The aim of our study was to identify potential lncRNA signatures capable of predicting overall survival (OS) of BC patients.

**Methods:**

The RNA sequencing data and clinical characteristics of BC patients were obtained from the Cancer Genome Atlas database, and differentially expressed lncRNA (DElncRNAs), DEmRNAs, and DEmiRNAs were then identified between BC and normal breast tissue samples. Subsequently, the lncRNA–miRNA–mRNA ceRNA network of BC was established, and the gene oncology enrichment analyses for the DEmRNAs interacting with lncRNAs in the ceRNA network was implemented. Using univariate and multivariate Cox regression analyses, a four-lncRNA signature was developed and used for predicting the survival in BC patients. We applied receiver operating characteristic analysis to assess the performance of our model.

**Results:**

A total of 1061 DElncRNAs, 2150 DEmRNAs, and 82 DEmiRNAs were identified between BC and normal breast tissue samples. A lncRNA–miRNA–mRNA ceRNA network of BC was established, which comprised of 8 DEmiRNAs, 48 DElncRNAs, and 10 DEmRNAs. Further gene oncology enrichment analyses revealed that the DEmRNAs interacting with lncRNAs in the ceRNA network participated in cell leading edge, protease binding, alpha-catenin binding, gamma-catenin binding, and adenylate cyclase binding. A univariate regression analysis of the DElncRNAs revealed 7 lncRNAs (ADAMTS9-AS1, AC061992.1, LINC00536, HOTAIR, AL391421.1, TLR8-AS1 and LINC00491) that were associated with OS of BC patients. A multivariate Cox regression analysis demonstrated that 4 of those lncRNAs (ADAMTS9-AS1, LINC00536, AL391421.1 and LINC00491) had significant prognostic value, and their cumulative risk score indicated that this 4-lncRNA signature independently predicted OS in BC patients. Furthermore, the area under the curve of the 4-lncRNA signature associated with 3-year survival was 0.696.

**Conclusions:**

The current study provides novel insights into the lncRNA-related ceRNA network in BC and the 4 lncRNA biomarkers may be independent prognostic signatures in predicting the survival of BC patients.

**Electronic supplementary material:**

The online version of this article (10.1186/s12967-018-1640-2) contains supplementary material, which is available to authorized users.

## Background

Breast cancer (BC) is a heterogeneous and malignant neoplasm derived from breast tissue, and accounts for about 16% of all cancers and 22.9% of invasive cancers in women [1]. The most common cause of the BC-related mortality is metastasis [2, 3]. Currently, BC diagnosis and prognosis is evaluated on the basis of disease stage, histological grade, and the expression level of hormone receptors [4]. However, clinical and pathological symptoms have limited predictive value in detecting early BC, and the clinical outcomes are highly variable on account of its heterogeneity. In addition, the underlying molecular mechanisms of BC still remain unclear. Therefore, it is vital to identify potential molecular diagnostic markers and/or therapeutic targets to combat BC, especially the invasive form.

Long non-coding RNAs (lncRNAs) is a class of ncRNA over 200 nucleotides long [5], and are reportedly involved in a number of cellular processes, for example, transcriptional and post-transcriptional regulation [5, 6]. Due to their strong tissue specificity, lncRNAs are potentially effective early diagnostic biomarkers of various cancers [7]. Identification of a BC specific lncRNA biomarker may therefore be of clinical significance for the diagnosis and prognosis of BC. Several lncRNAs have been reported to be associated with BC initiation and progression [8, 9], and although some have been found to predict clinical outcomes for BC, the results are inconsistent due to limited tissue samples. Furthermore, studies without large sample size are also not able to determine with statistical power whether these lncRNAs are associated with survival or other clinical factors. The Cancer Genome Atlas (TCGA) is an open-access and large-scale database which can provide multidimensional molecular profiles for a large number tumor samples. To increase the statistical reliability of our studies, we identified BC specific lncRNAs using data obtained from TCGA database.

The competing endogenous RNA (ceRNA) hypothesis presented by Salmena et al. [10] was proposed as a novel regulatory mechanism between ncRNA and coding messenger RNA. LncRNAs contain miRNA-response elements (MREs) which function as ceRNAs, and play a key role in various pathological processes like tumorigenesis [11]. Zhang et al. [12] have proved the biological role of lncRNA related-ceRNAs in glioblastomas. A recent study has demonstrated that lncRNA NUTF2P3-001 acts as a ceRNA to communicate with KRAS by competitively binding to hsa-mir-3923, and the up-regulation of NUTF2P3-001 reverses the suppressive effect of hsa-mir-3923 on KRAS, leading to the proliferation and invasion of pancreatic cancer [13]. In addition, the aberrant expression of 7-lncRNA signature (called LncRisk-7) led to differential gene expression via a dysregulated lncRNA-associated ceRNA network, contributing to pancreatic ductal adenocarcinoma progression [14]. Collectively, these findings show that dysregulation of important lncRNAs in the ceRNA network also disrupt the miRNA-mediated lncRNA/mRNA ceRNA interactions and therefore contribute to cancer initiation and progression [15, 16]. Nevertheless, very little information is available on BC ceRNAs.

In our work, RNA sequencing data of 1109 BC samples and 113 adjacent non-tumor breast tissues samples were retrieved from the TCGA database. To the best of our knowledge, this is the first study to use large scale sequencing database (TCGA) and ceRNA network to identify BC-specific lncRNAs. This new approach of predicting cancer specific lncRNA and ceRNA networks can elucidate the lncRNA-mediated ceRNA regulatory mechanisms in the development and prognosis of BC, and identify novel lncRNAs as potential diagnostic biomarkers or therapeutic targets.

## Methods

### Patients and samples from the TCGA database

RNA sequencing (RNA-Seq) data associated with BC were retrieved from the TCGA database (https://portal.gdc.cancer.gov/, version 10.1, release time: February 15, 2018). A total of 1222 individuals with BC were included in the current study. The exclusion criteria were (1) histological diagnosis negating BC, (2) presence of a malignancy other than BC, and (3) lack of complete clinical data. The gene expression profiles of 1109 BC and 113 adjacent normal breast tissues, and miRNA data of 1103 BC and 104 adjacent normal breast tissues were downloaded. In addition to the RNA expression data, clinical information of BC patients were also downloaded from the TCGA database. No approval from the ethics committee was needed because all the information was required from the TCGA database. The clinical characteristics for BC patients are listed in Table 1.

**Table 1:** The predictive values of clinical features and risk score

Variables	Patients (N)	Univariate analysis		Multivariate analysis	
		HR (95% CI)	*P*	HR (95% CI)	*P*
Age					
< 60/≥ 60	572/506	1.67 (1.13–2.48)	0.010	2.46 (1.37–4.42)	0.003
Pathologic stage					
I–II/III–IV	799/279	2.47 (1.67-–3.66)	0.000	2.86 (1.15–7.11)	0.024
Stage T					
T1–T2/T3–T4	901/177	1.26 (0.8–1.97)	0.323	1.41 (0.59–3.35)	0.440
Stage N					
N0/NX	512/566	2.56 (1.65–3.95)	0.000	1.12 (0.53–2.37)	0.773
Stage M					
M0/MX	901/177	1.79 (1.05–3.07)	0.034	0.48 (0.17–1.33)	0.156
ER					
Negative/positive	232/796	0.58 (0.38–0.88)	0.011	1.15 (0.31–4.32)	0.837
PR					
Negative/positive	338/687	0.57 (0.38–0.86)	0.007	0.54 (0.2–1.47)	0.226
Her2					
Negative/positive	552/163	2.3 (1.29–4.1)	0.005	2.24 (1.06–4.72)	0.034
Triple negative					
No/yes	598/112	1.59 (0.82–3.07)	0.168	1.69 (0.46–6.19)	0.428
Risk score					
Low/high	593/593	2.16 (1.42–3.29)	0.000	2.26 (1.15–4.43)	0.017

*HR* hazard ratio, *CI* confidence interval

### RNA sequence data processing and differential expression analysis

The raw RNA sequencing (lncRNA, miRNA, and mRNA) reads were post-processed and normalized using the trimmed mean of M-values (TMM) method.

EdgeR package in R (version 3.4.1) was used to identify the differentially expressed mRNAs (DEmRNAs), lncRNAs (DElncRNAs) and miRNAs (DEmiRNAs) between the BC and adjacent-normal breast tissues [17], and the cut-off criteria were set as P < 0.01 and |logFC| > 2. Volcano plots were visualized using the ggplot2packages in R [18]. The heat map was plotted using the pheatmap function of pheatmap package version 1.0.8 [19].

### Establishment of the ceRNA network

The lncRNA–miRNA–mRNA ceRNA network was constructed based on the hypothesis that lncRNAs directly interact with and regulate the activity of mRNAs by acting as miRNA sponges [20]. Based on this hypothesis, we established the lncRNA–miRNA–mRNA ceRNA network in three steps: (1) BC-specific RNAs (lncRNA, mRNA, and miRNA) with P < 0.01, and |logFC| > 2 were reserved, (2) the potential miRNAs targeted by DElncRNAs and the lncRNA–miRNA interactions were predicted by the miRcode online tool (http://www.mircode.org), and (3) the MiRDB (http://www.mirdb.org/), miRTarBase (http://mirtarbase.mbc.nctu.edu.tw//), and Targetscan (http://www.targetscan.org//) programs were used to predict the target mRNAs of miRNAs. Finally, the miRNAs that were negatively regulated by the lncRNAs and mRNAs were selected to build the ceRNA network. Cytoscape (version 3.5.1) was used to visualize the lncRNA–miRNA–mRNA ceRNA network.

### Functional enrichment analysis

Gene oncology (GO) is widely used as functional enrichment analysis for a large number of genes [21]. The putative biological roles of DElncRNAs corresponds to that of their associated mRNAs. GO function analyses were therefore conducted for the DEmRNAs in the ceRNA network using R clusterProfiler package [22]. Fisher’s test was used to identify the significant GO terms, and GO categories with P < 0.05 were considered statistically significant.

### Construction of the BC-specific prognostic signatures

Kaplan–Meier and log-rank test was used to determine the association between the DEmRNAs, DElncRNAs and DEmiRNAs in the ceRNA network and the overall survival (OS) of BC patients, and statistical significance was set at P < 0.05. Univariate Cox proportional hazards regression method was implemented to analyze the relationship between the DElncRNAs and OS when a significant level was set at 0.05, in order to determine those with a prognostic value in BC. This was followed by multivariate Cox hazards regression model to determine the independent prognostic factors for BC, and the prognostic risk score for predicting OS was as follows:

$${\text{Risk}} \;{\text{score}} = \exp_{\text{lncRNA1}} * \;\upbeta_{{_{\text{lncRNA1}} }} + \exp_{\text{lncRNA2}} *\; \upbeta_{{_{\text{lncRNA2}} }} + \cdots \exp_{\text{lncRNAn}} *\; \upbeta_{{_{\text{lncRNAn}} }}$$

(where “exp” denotes the expression level of DElncRNAs, and “β” is the regression coefficient obtained from the multivariate Cox regression model) [23]. Using the median risk score as the threshold, the BC patients were stratified into the high- and low-risk groups. The “survival ROC” package in R was used to construct the time-dependent receiver operating characteristic (ROC) curves within 3 years as the defining point, and to measure the risk prediction rate of specific lncRNAs between the two groups. In addition, the univariate and multivariate analyses were used to evaluate the effects of other clinical variables of BC patients on OS risk scores. The R software (version 3.4.1) was used for all statistical analyses.

## Results

### Identification of DEmRNAs, DElncRNAs, and DEmiRNAs

**Fig. 1:**
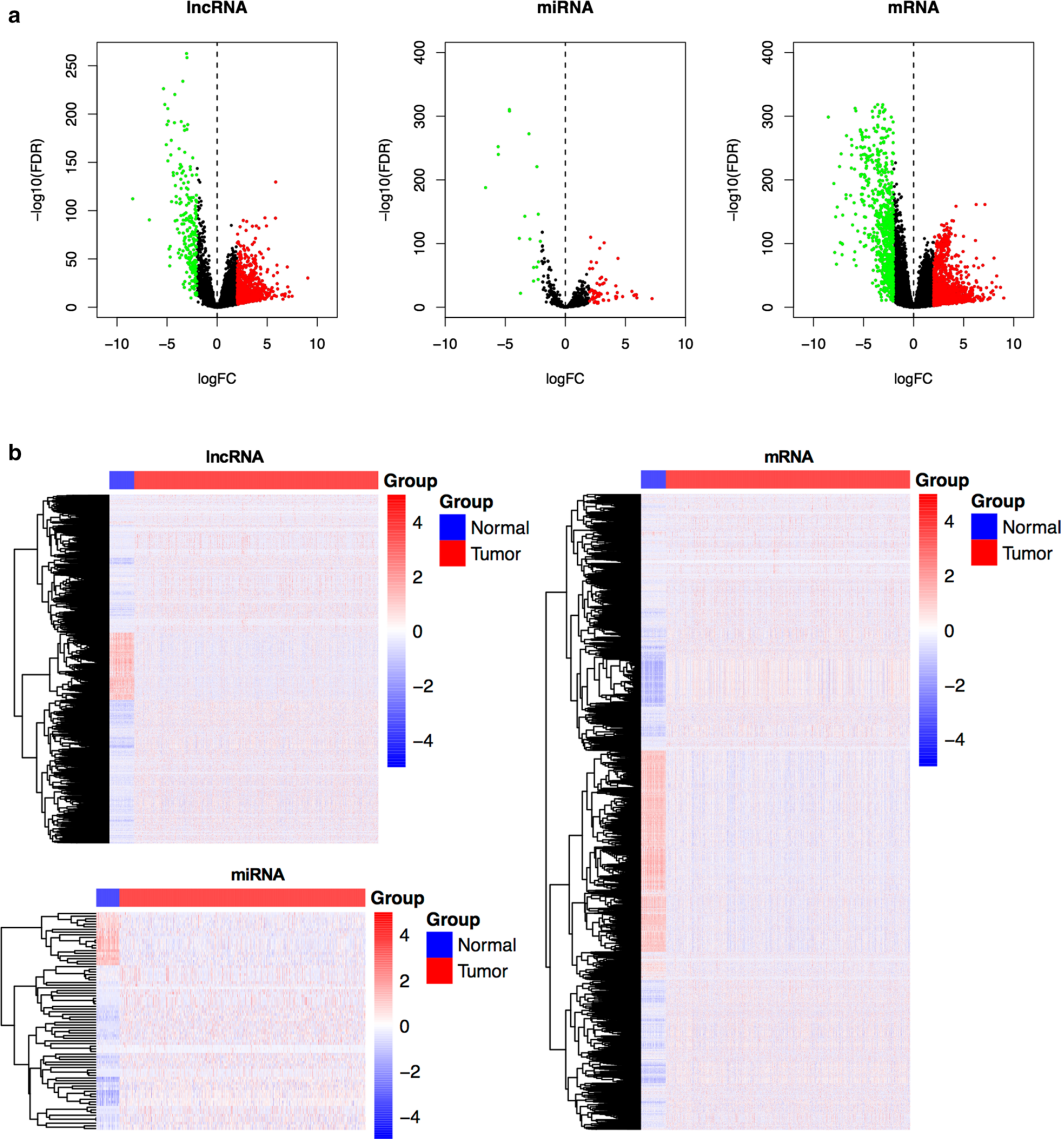
**a** Volcano plots showing the differential expression of RNAs (lncRNAs, miRNAs, and mRNAs) in breast cancer (BC), which were drawn using the R packages ggplot2; X axis indicates the mean expression differences of lncRNAs, miRNAs, and mRNAs between BC and normal samples, and Y axis represents log transformed false discovery rate (FDR) values. **b** Heatmaps demonstrate differential expression of lncRNAs, miRNAs, and mRNAs between BC and adjacent normal samples, which were plotted using the pheatmap package; X axis denotes differentially expressed lncRNAs (DElncRNAs), DEmiRNAs, and DEmRNAs and Y axis represents the samples. Blue represents normal samples, while red stands for BC samples. The expression values are shown in line with the color scale

We identified the DEmRNAs, DElncRNAs, and DEmiRNAs in BC and adjacent-normal breast tissues using the TCGA database, with P < 0.01 and |logFC| > 2 as the thresholds. A total of 2150 DEmRNAs (1368 up- and 782 down-regulated), 1061 DElncRNAs (839 up-, and 222 down-regulated), and 82 DEmiRNAs (62 up- and 20 down-regulated) were identified between BC and normal samples. Volcano plots displaying the distribution of the DElncRNAs, DEmiRNAs, and DEmRNAs were generated, as shown in Fig. 1a. The heat map showed clear separation and consistency in the expression profiles of the BC and normal samples (Fig. 1b).

### MiRNA predicted target analysis and ceRNA network establishment

The differentially expressed RNAs identified above were selected, and the lncRNAs and mRNAs targeted by miRNAs were extracted to establish the lncRNA–miRNA–mRNA ceRNA network. The relationships among 1061 DElncRNAs and 82 DEmiRNAs were first evaluated. Since lncRNAs might interact with the miRNAs through MREs, the miRcode tool was then used to detect the potential MREs; 18 BC-specific miRNAs that putatively target 70 BC-specific lncRNAs were then identified (Additional file 1: Table S1). The MiRDB, miRTarBase and Targetscan programs were then used to determine the relationship between the 82 DEmiRNAs and 2150 DEmRNAs, and predict the mRNA targets of miRNAs. The results indicated that 8 BC specific miRNAs targeted 10 BC-specific mRNAs (Additional file 1: Table S2).

**Fig. 2:**
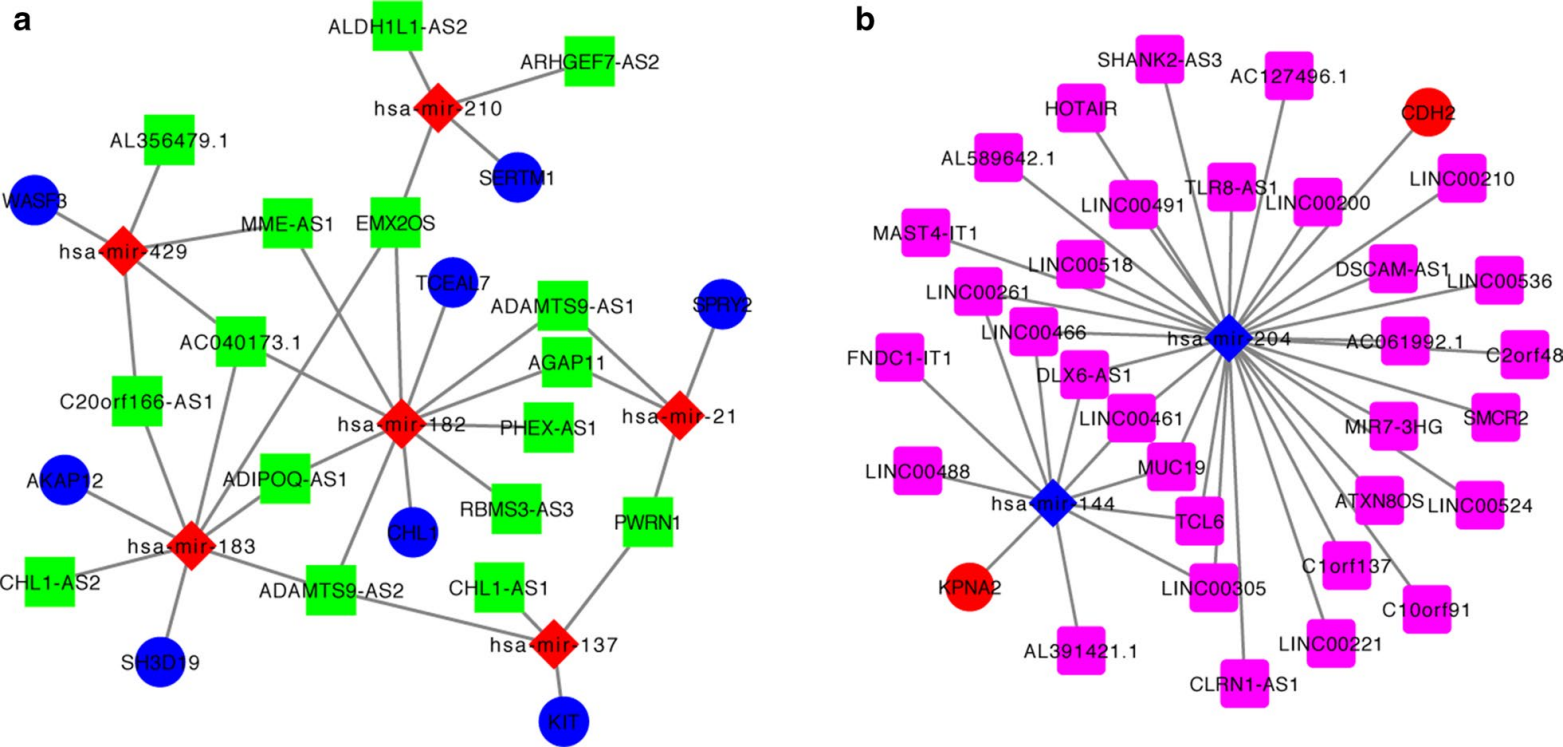
LncRNA-miRNA-mRNA competing endogenous RNA (ceRNA) network of under-expressed (**a**) and over-expressed (**b**) lncRNAs–mRNAs. In network **a** blue circles = down-regulated mRNAs, green rectangle = down-regulated lncRNAs, red diamonds = up-regulated miRNAs. In network **b** red circles = up-regulated mRNAs, pink rectangle = up-regulated lncRNAs, and blue diamonds = down-regulated miRNAs

On the basis of the above data, the lncRNA–miRNA–mRNA ceRNA network was established and plotted using Cytoscape 3.5.1. Overall, 8 miRNAs (6 up-, and 2 down-regulated, Table 2), 48 lncRNAs (32 up-, and 16 down-regulated, Table 3), and 10 mRNAs (2 up-, and 8 down-regulated, Table 4) were involved in the proposed ceRNA network (Fig. 2). Based on the expression levels of DEmRNAs, DElncRNAs and DEmiRNAs, two ceRNA networks including under-expressed (Fig. 2a) and over-expressed (Fig. 2b) networks were constructed.

**Table 2:** miRNAs in ceRNA network of BC

miRNA	logFC	*P* value	FDR
hsa-mir-210	3.022945333	5.83E−49	7.72E−48
hsa-mir-183	2.861143375	8.29E−95	2.30E−93
hsa-mir-429	2.590469156	3.89E−72	7.09E−71
hsa-mir-137	2.481127225	4.30E−10	9.52E−10
hsa-mir-182	2.265833003	1.04E−62	1.64E−61
hsa-mir-21	2.105244174	2.87E−112	1.19E−110
hsa-mir-204	− 2.643779105	2.00E−64	3.23E−63
hsa-mir-144	− 2.948045408	2.31E−109	8.42E−108

**Table 3:** lncRNA in ceRNA network of BC

lncRNA	logFC	*P* value	FDR
DSCAM-AS1	5.99941085	4.44E−26	4.13E−25
LINC00261	5.43693494	3.30E−10	9.98E−10
LINC00305	5.05852227	2.85E−13	1.14E−12
ATXN8OS	4.46010816	3.17E−11	1.06E−10
LINC00221	4.39970195	1.02E−12	3.88E−12
LINC00210	4.30883058	4.11E−11	1.36E−10
LINC00466	4.24876959	2.08E−59	7.81E−58
LINC00518	4.15626859	1.43E−15	6.88E−15
MIR7-3HG	4.10845802	9.51E−14	3.95E−13
MUC19	4.07948976	3.60E−20	2.36E−19
LINC00461	3.60834524	3.96E−19	2.43E−18
AL589642.1	3.54021424	2.88E−10	8.74E−10
C2orf48	3.44559688	6.13E−56	2.05E−54
LINC00200	3.39237322	5.31E−09	1.43E−08
AL391421.1	3.26256052	3.52E−28	3.66E−27
HOTAIR	2.99777460	1.71E−29	1.92E−28
CLRN1-AS1	2.96319950	3.12E−29	3.42E−28
SMCR2	2.94279962	3.39E−45	7.75E−44
TCL6	2.90585029	2.21E−21	1.57E−20
LINC00536	2.82907445	1.74E−33	2.43E−32
LINC00524	2.61155065	7.48E−15	3.41E−14
LINC00488	2.59389493	3.24E−08	8.12E−08
FNDC1-IT1	2.36336842	3.22E−22	2.41E−21
C10orf91	2.33148238	1.26E−24	1.09E−23
MAST4-IT1	2.28036554	1.05E−07	2.51E−07
TLR8-AS1	2.26971966	2.22E−11	7.45E−11
C1orf137	2.26365184	3.59E−12	1.29E−11
AC061992.1	2.24485511	7.75E−45	1.75E−43
SHANK2-AS3	2.15540014	1.36E−08	3.53E−08
AC127496.1	2.09598212	6.09E−30	7.02E−29
LINC00491	2.05604565	7.95E−06	1.59E−05
DLX6-AS1	2.019341904	6.94E−11	2.25E−10
EMX2OS	− 2.12877927	6.46E−83	4.40E−81
PWRN1	− 2.18494962	4.42E−16	2.21E−15
CHL1-AS1	− 2.32544868	1.99E−39	3.63E−38
C20orf166-AS1	− 2.40983541	4.19E−43	8.75E−42
PHEX-AS1	− 2.41002550	4.36E−29	4.73E−28
AL356479.1	− 2.47223771	1.01E−37	1.72E−36
RBMS3-AS3	− 2.55564241	5.60E−113	8.32E−111
AGAP11	− 2.57967944	1.99E−116	3.36E−114
AC040173.1	− 2.62010067	2.11E−41	4.18E−40
ADAMTS9-AS1	− 2.65113038	4.59E−96	4.63E−94
ARHGEF7-AS2	− 2.77120520	1.03E−114	1.61E−112
CHL1-AS2	− 2.80546689	7.46E−76	4.28E−74
MME-AS1	− 3.01455968	2.41E−62	9.89E−61
ADAMTS9-AS2	− 3.02086308	1.16E−187	7.25E−185
ALDH1L1-AS2	− 4.86910828	2.30E−196	2.53E−193
ADIPOQ-AS1	− 5.07515076	1.06E−171	5.48E−169

**Table 4:** mRNA in ceRNA network of BC

mRNA	logFC	*P* value	FDR
CDH2	2.57235331	7.09E−27	3.37E−26
KPNA2	2.14681192	3.85E−77	7.06E−76
SH3D19	− 2.0172533	2.91E−244	4.34E−242
TCEAL7	− 2.1550474	8.72E−128	3.65E−126
WASF3	− 2.1800702	6.27E−121	2.38E−119
SPRY2	− 2.4420515	6.19E−188	5.38E−186
AKAP12	− 2.7016705	6.57E−188	5.69E−186
CHL1	− 2.9649624	5.86E−121	2.23E−119
KIT	− 2.9705757	7.12E−126	2.85E−124
SERTM1	− 3.6706976	2.69E−84	5.55E−83

### Delineation of GO analysis

In order to better understand the role of the DElncRNAs in BC, we analyzed the mRNAs of the ceRNA network and identified the lncRNA regulated GO terms. The results of GO analysis are shown in Additional file 2: Figure S1. Our data showed that the mRNAs associated to cellular component (CC), was cell leading edge. Ge et al. [24] have demonstrated that trypsin secreted from MDA MB-231 BC cells activates the protease-activated receptor-2 and the activated protease-activated receptor-2 can promote cell migration based on ERK1/2-dependent pathway, involving the formation of a scaffolding complex at the cell leading edge. Meanwhile, the mRNAs related to molecular function (MF) were most relevant to protease binding, alpha-catenin binding, gamma-catenin binding, and adenylate cyclase binding. Proteases provide the cancer a characteristic of being able to invade into other tissues, and protease-activated receptor-1 is involved in the migration and invasion of breast cancer cells [25]. The loss of alpha-catenin has been implicated to be related to the metastasis and poor survival in BC [26]. The expression of gamma-catenin was also reported to be associated with the metastasis in human BC [27]. The activity of the adenylate cyclase is positively linked to the inhibition of cell proliferation, as well as induction of apoptosis in human BC MCF-7 cells [28]. Demonstrated herein, these GO terms are associated with BC pathogenesis and prognosis.

### Correlations between BC specific signatures and OS

**Fig. 3:**
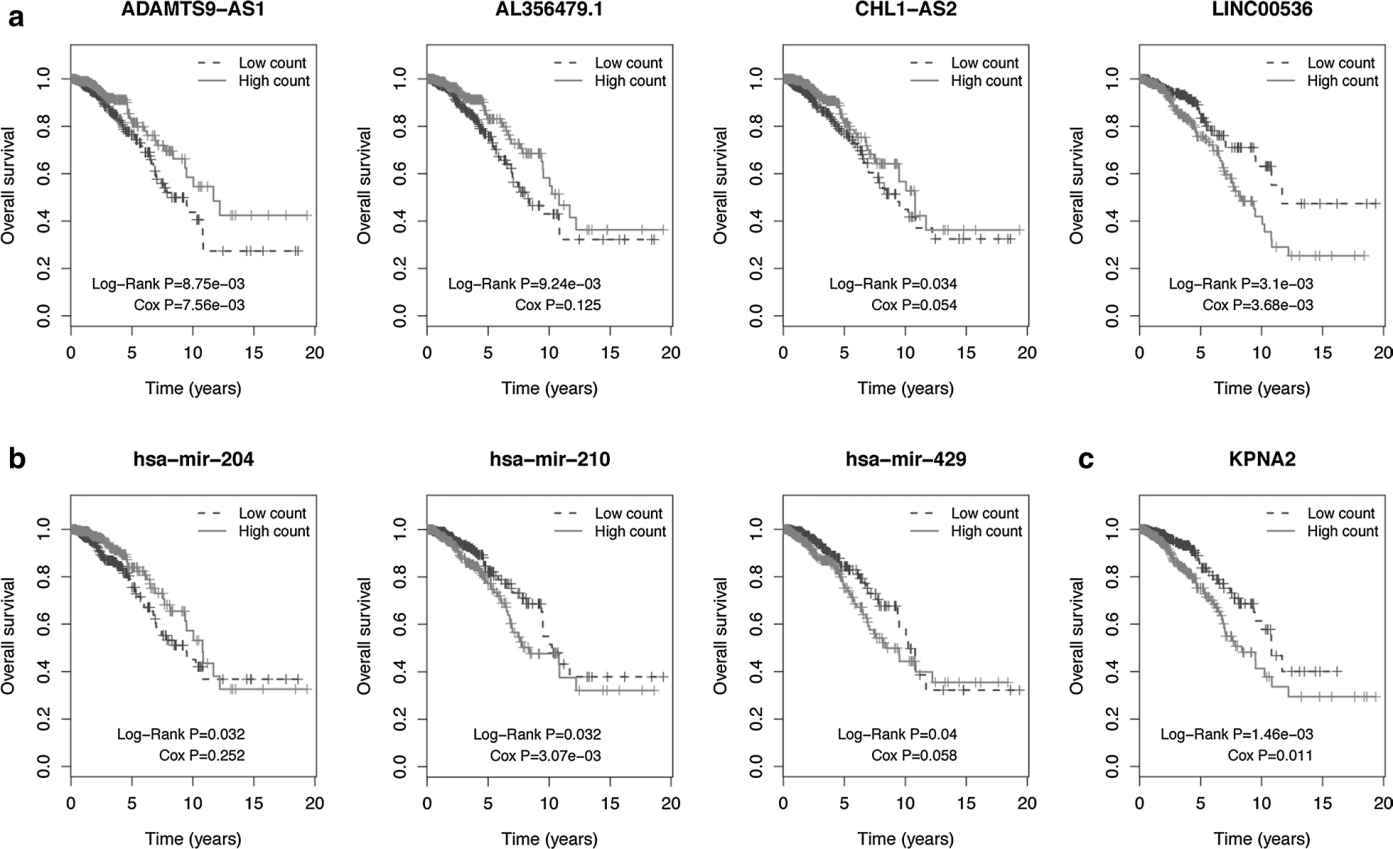
Kaplan–Meier survival curves for **a** DElncRNAs, **b** DEmiRNAs, and **c** DEmRNAs associated with overall survival (OS) of the BC patients. Log-rank method was used to assess the survival differences between the two groups. Horizontal axis is OS time (years) and vertical axis stands for survival function

Kaplan–Meier and log-rank test were used to determine the relationship between the DEmRNAs, DElncRNAs and DEmiRNAs in the ceRNA network and the OS of BC patients with a cut-off threshold of P < 0.05. Totally, 4 DElncRNAs (ADAMTS9-AS1, AL356479.1, CHL1-AS2, and LINC00536; Fig. 3a), 3 DEmiRNAs (hsa-miR-204, hsa-miR-210, and hsa-miR-429; Fig. 3b), and 1 DEmRNA (KPNA2; Fig. 3c) were found to be related to OS.

### Establishment of the 4-lncRNAs prognostic model

Univariate regression analysis was used to identify the lncRNAs associated with the OS of BC patients. With the significance level cutoff threshold set at P < 0.05, a group of lncRNA signatures including ADAMTS9-AS1, AC061992.1, LINC00536, HOTAIR, AL391421.1, TLR8-AS1, and LINC00491 lncRNAs was detected to have significant prognostic value (Additional file 1: Table S3). Significantly, we found that ADAMTS9-AS1 was simultaneously identified to be connected with OS in Kaplan–Meier (log-rank test) and univariate Cox regression analysis.

All the above lncRNAs were then fitted into the multivariate Cox regression model, which indicated that only four lncRNAs—ADAMTS9-AS1, LINC00536, AL391421.1 and LINC00491—had a significant prognostic value in BC (Additional file 3: Figure S2C), and these four lncRNAs were used to develop an lncRNA prognostic model. A risk score analysis of the four lncRNAs was performed for each patient, and based on the risk scores, the patients were divided into the “low risk” and “high risk” groups (Additional file 3: Figure S2A). The mortality rate of the high risk patients was significantly higher compared to the low risk patients (12.99% vs 5.94%; P < 0.05; Additional file 3: Figure S2B).

**Fig. 4:**
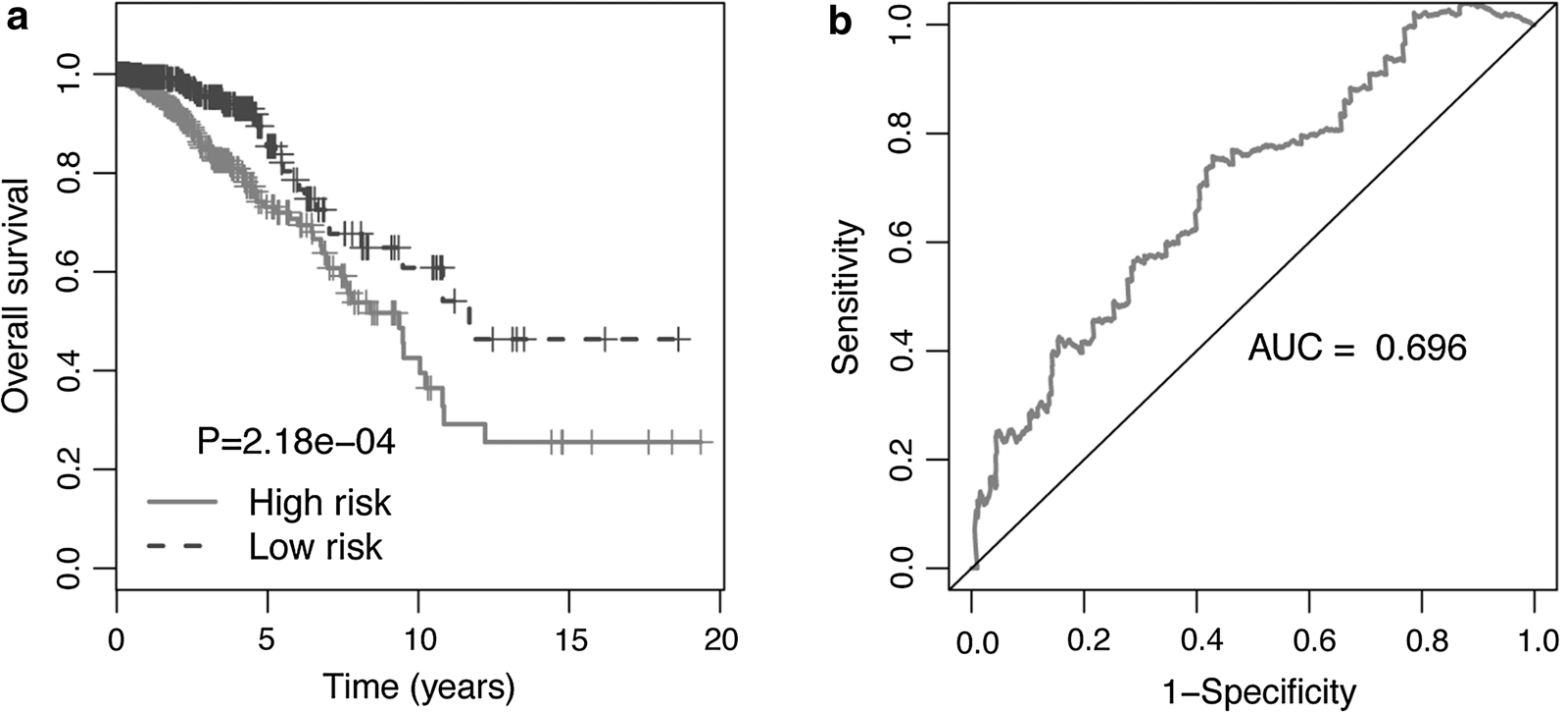
The 4-lncRNA signature of BC (ADAMTS9-AS1, AL391421.1, LINC00491, and LINC00536) for the outcome. **a** The survival differences between the high-risk and low-risk groups were determined by the log-rank test (P = 2.18E−04). **b** ROC curves demonstrated that the area under receiver operating characteristic (AUC) of 4-lncRNA model was 0.696, which exhibited the risk score

In addition, the high risk group was correlated with worse prognosis compared to the low risk group (Fig. 4a). The 3 year survival correlation of the 4-lncRNA signature was analyzed by ROC and AUC was computed to assess the discriminatory capacity of lncRNA signature (Fig. 4b). The AUC of the 4-lncRNA signature was 0.696 indicating its utility as a prognostic model for predicting the survival status of BC.

**Fig. 5:**
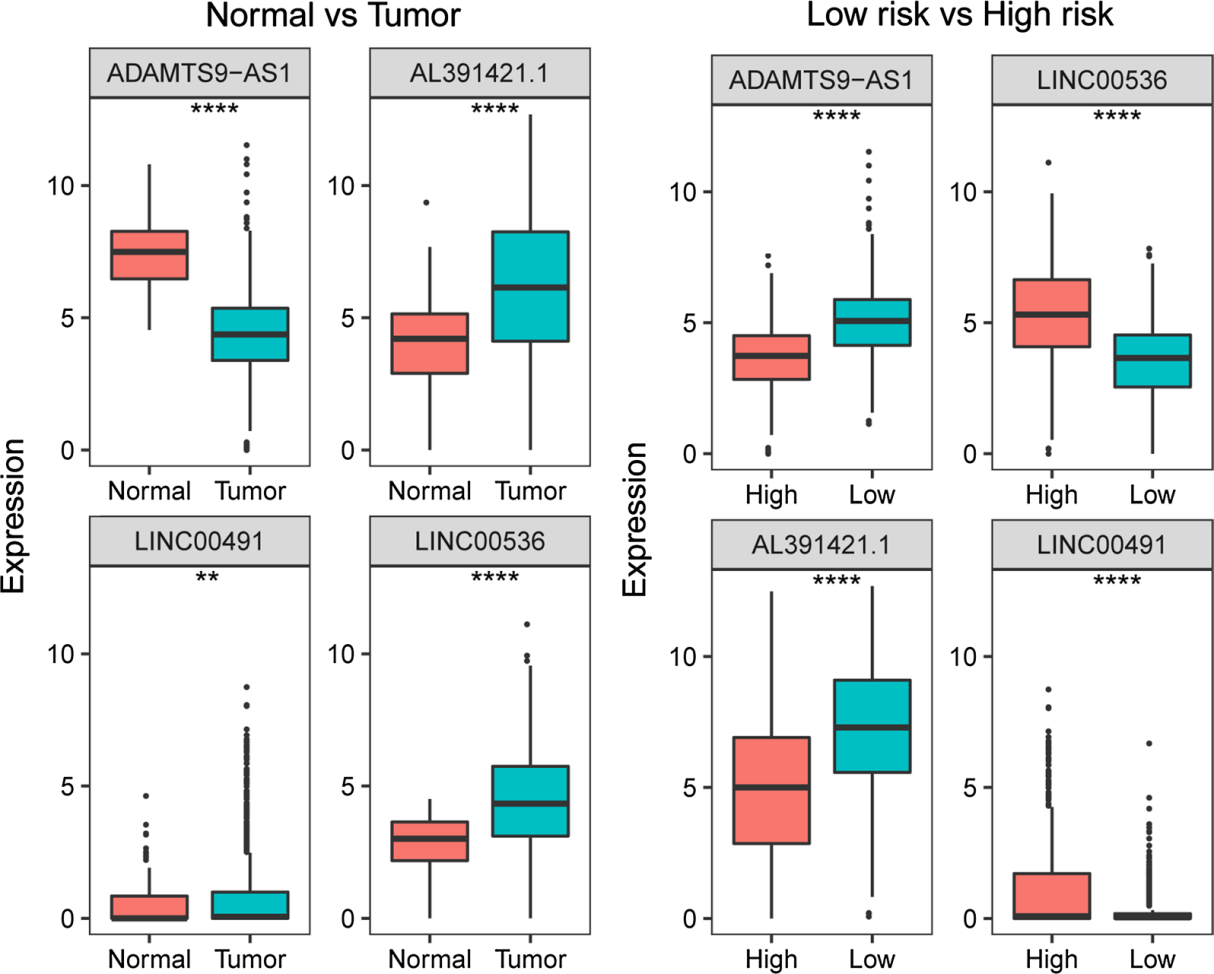
Expression pattern of the 4-lncRNA (ADAMTS9-AS1, AL391421.1, LINC00491, and LINC00536) in BC and normal tissues, and in high-risk and low-risk groups

The 4-lncRNA expression was then analyzed in the tumor and normal tissues, and in the high- and low-risk patient groups (Fig. 5). ADAMTS9-AS1, and AL391421.1 were expressed at high levels in patients with low-risk scores, whereas LINC00536 and LINC00491 were up-regulated in the high-risk patients. Furthermore, LINC00491, AL391421.1, and LINC00536 were expressed at high levels, and ADAMTS9-AS1 was expressed at low levels in the BC patients.

### Prognostic value of the four-lncRNA signature in BC

**Fig. 6:**
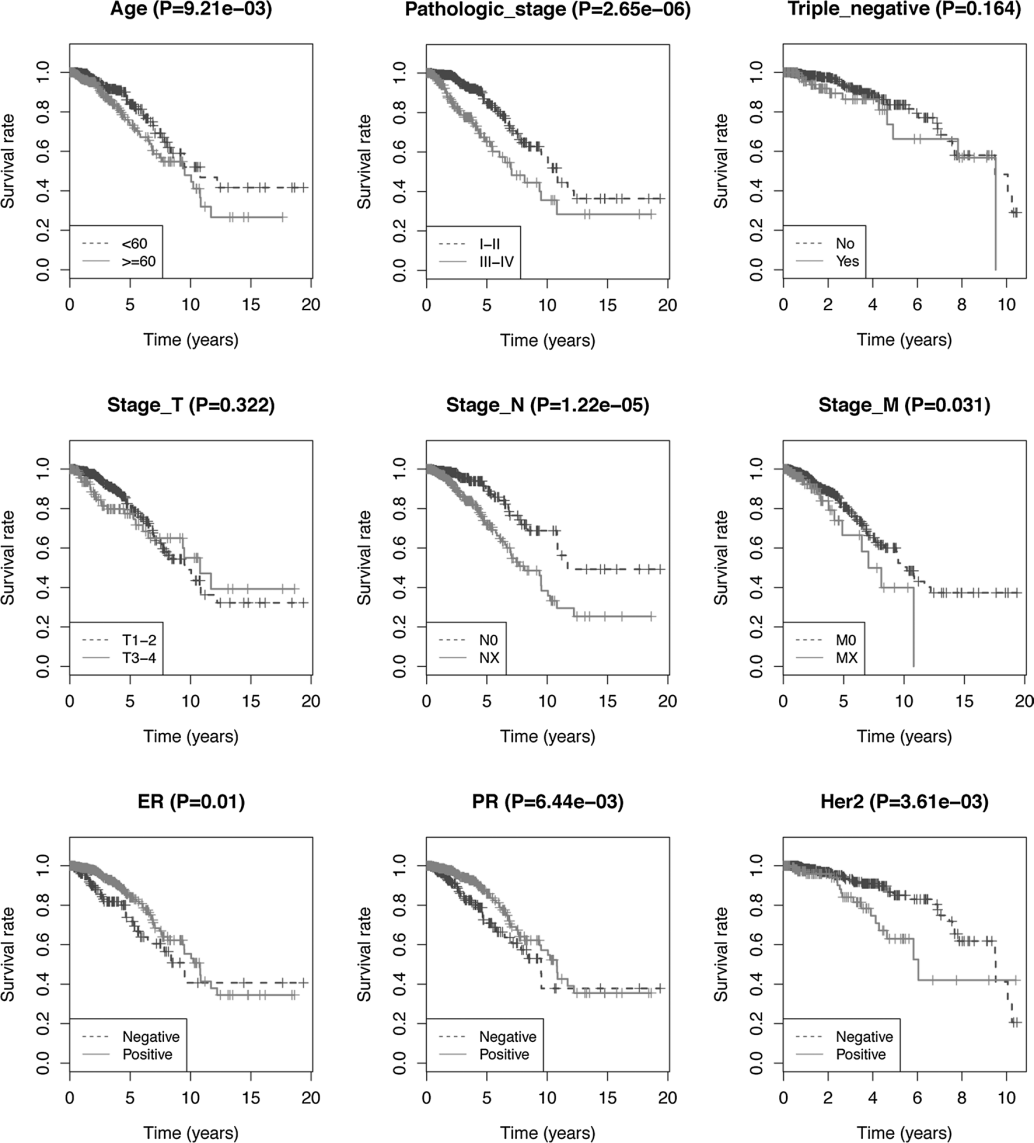
Kaplan–Meier curves utilized to compare the survival when patients were stratified by clinical characteristics (age, pathologic stage, stage N, stage M, ER, PR, Her2 and so on)

Univariate and multivariate regression models were used to assess the prognostic power of the 4-lncRNA signature. Univariate analysis indicated that age, pathological stage, N stage, M stage, ER, PR, Her2, and risk scores were significantly correlated with OS of BRCA patients (P < 0.05). Similarly, Kaplan–Meier analysis demonstrated that clinical factors (age, pathological stage, N stage, M stage, ER, PR, and Her2) were significantly correlated to OS, which was consistent with the univariate analysis. Kaplan–Meier curves of the clinical characteristics are shown in Fig. 6. Multivariate analysis indicated that only age, pathological stage, Her2, and risk scores were independent prognostic factors of OS (P= 0.003, 0.024, 0.034, and 0.017 respectively; Table 1).

## Discussion

BC is a common malignant gynecological cancer, and is one of the main causes for the cancer-related deaths in women [29]. The lack of specific diagnostic and prognostic biomarkers may contribute to the current low survival rate among BC patients. To improve clinical outcomes therefore, it is essential to explore the exact regulatory mechanisms of BC initiation and progression, and to identify the potential BC-related prognostic signatures that predict those outcomes. Growing experimental evidence indicates that lncRNAs play important roles in many biological processes, and ceRNA activity is closely related to the development of cancers [30, 31].

In recent years, some studies have investigated the ceRNAs in BC. For instance, Chen et al. [32] analyzed the BC ceRNA network on the basis of common miRNAs as well as co-expression, but did not consider miRNA expression. Another study also established a BC specific ceRNA network to investigate its underlying molecular mechanisms based on the PCC of miRNA–mRNA pairs [33]. However, both studies focused on the roles of mRNAs rather than that of lncRNAs in the BC ceRNA networks. In 2018, Zhou et al. [34] constructed four BC-related ceRNA networks by combining the miRNA targets and the expression data of lncRNAs, miRNAs and mRNA, but they did not take into account the relationship between survival and lncRNAs, nor construct the prognostic signature. In our study, in addition to constructing the ceRNA networks by combining lncRNA, miRNA, and mRNA expression data, we also investigated the association of lncRNA and OS in BC patients. Furthermore, based on the theory of ceRNA network, we established the 4-lncRNAs prognostic signature. With the goal of identifying lncRNAs significantly associated with OS, we established an lncRNA–miRNA–mRNA ceRNA network using the information obtained from the TCGA database. Univariate regression analysis on the DElncRNAs of the ceRNA network identified 7 lncRNAs—ADAMTS9-AS1, AC061992.1, LINC00536, HOTAIR, AL391421.1, TLR8-AS1 and LINC00491—that were associated with OS. Multivariate analysis showed significant prognostic value of 4 of those lncRNAs (ADAMTS9-AS1, LINC00536, AL391421.1 and LINC00491) in the OS of BC patients. A cumulative risk score of the 4 lncRNAs was calculated, which indicated that this 4-lncRNA signature independently predicted OS in BC patients. To the best of our knowledge, this is the first report integrating a ceRNA network with TCGA data to build an lncRNA-related risk score, and evaluate the OS of BC patients. Our study will help improve the understanding of lncRNA-mediated ceRNA regulatory mechanisms in BC and identify novel lncRNAs as therapeutic targets.

In the current study, among this 4-lncRNA signature, ADAMTS9-AS1 was demonstrated to play important roles in the progression and prognosis of cancer. ADAMTS9-AS1 is an antisense lncRNA, and growing evidence has implicated that a large amount of antisense lncRNAs play crucial roles in the cancer [35, 36]. Li et al. [37] reported that ADAMTS9-AS1 could predict the survival status of patients with esophageal squamous cell carcinoma. Another study has also indicated a prognostic role of ADAMTS9-AS1 in patients with colon adenocarcinoma [38]. In addition, ADAMTS9-AS1 has been demonstrated to be a risk lncRNA in ovarian cancer, which is involved in the progression of ovarian cancer [39]. In our study, we noticed that ADAMTS9-AS1 with low-expression could compete with up-regulated miRNAs (hsa-mir-182, and hsa-mir-21), to regulate the expression of the target genes such as CHL1, SPRY2, and TCEAL7 involved in the ceRNA network. Previous studies have shown high expression of hsa-mir-182 in MCF-7 breast cancer cells [40, 41]. In addition, the high-expression of hsa-mir-21 was reported to be correlated to the metastasis and poor prognosis of BC patients [42]. The remaining three lncRNAs of the ceRNA network (LINC00536/AL391421.1/LINC00491) were up-regulated and competed with the decreased hsa-mir-204 and hsa-mir-144 levels. Down-regulation of has-mir-204 has been suggested to enhance cell proliferation and invasion in gastric cancer [43], and low-expression of has-mir-204 is related to the poor prognosis of acute myeloid leukemia patients [44]. In addition, decreased expression of has-mir-144 is strongly correlated with the progression of colorectal cancer [45]. No study so far has reported any association of LINC00536, AL391421.1 or LINC00491 with cancer. This is the first study to show aberrant expression of ADAMTS9-AS1, LINC00536, AL391421.1 and LINC00491 in BC, and indicates a potential prognostic role of this 4-lncRNA signature in BC. In addition, the bioinformatics based investigation of lncRNAs will be helpful in future experimental studies.

Although the findings of our study have important clinical implications, the limitations must also be noted. First, a longer follow-up duration is required to verify our results, and second, the findings based on the TCGA database will need to be verified using other experimental methods. In addition, the biological roles of ADAMTS9-AS1, LINC00536, AL391421.1, and LINC00491 in BC also need to be further investigated.

## Conclusion

Taken together, we have identified a 4-lncRNA signature as a potential prognostic predictor for BC patients by analyzing the genome-wide lncRNA expression data from the TCGA database based on a ceRNA network. The current findings provide novel insights into the lncRNA-related ceRNA network in BC and identify potential diagnostic and prognostic biomarkers. Further functional studies are needed to elucidate the molecular mechanisms underlying lncRNA function in BC.

## Additional files

**Additional file 1.** miRNAs targeting lncRNAs and mRNAs ofBC, as well as the prognostic value of the lncRNAs obtained from the univariate Cox’s analysis.

**Additional file 2: Figure S1.** Gene Ontology (GO) analysis. GO results for aberrantly expressed mRNAs with significant Enrichment score covering domains of cellular components (CCs) and molecular functions (MFs). The bar plot devotes the enrichment scores of the significant GO terms.

**Additional file 3: Figure S2.** Four-lncRNA signature ((ADAMTS9-AS1, AL391421.1, LINC00491, and LINC00536)) predicted OS in BC cohort. A. Risk-score distribution. Red demonstrating higher expression while blue representing lower expression. Risk scores for all BC patients were created in ascending order and blue is marked as low risk or red is labeled as high risk. B. Patients’ survival status with blue devoting dead, and red standing for alive. C. Heat map of the four-lncRNA expression profiles in BC patients.

## Abbreviations

lncRNAs: long non-coding RNAs
ceRNAs: competing endogenous RNAs
BC: breast cancer
OS: overall survival
TCGA: the Cancer Genome Atlas
DElncRNAs: differentially expressed lncRNA
MREs: miRNA-response elements
GO: gene oncology
ROC: receiver operating characteristic
CC: cellular component:
MF: molecular function
