# Supplementary material for: Systematic analysis of lncRNA–miRNA–mRNA competing endogenous RNA network identifies four-lncRNA signature as a prognostic biomarker for breast cancer
Source: J Transl Med. 2018 Sep 27;16:264. doi: 10.1186/s12967-018-1640-2 (PMC6161429; doi:10.1186/s12967-018-1640-2)
Supplement: Supplementary file 1 — Additional file 1. miRNAs targeting lncRNAs and mRNAs ofBC, as well as the prognostic value of the lncRNAs obtained from the univariate Cox’s analysis. [file 12967_2018_1640_MOESM1_ESM.docx]

**Table S1 miRNAs targeting lncRNAs of BC**

| **lncRNA** | **miRNA** |
| --- | --- |
| AGAP11 | hsa-mir-141, hsa-mir-200a, hsa-mir-182, hsa-mir-21, hsa-mir-375 |
| C20orf166-AS1 | hsa-mir-301b, hsa-mir-183, hsa-mir-429, hsa-mir-375 |
| AC135178.1 | hsa-mir-122 |
| AL356479.1 | hsa-mir-429 |
| PHEX-AS1 | hsa-mir-301b, hsa-mir-96, hsa-mir-182, hsa-mir-122 |
| CHL1-AS2 | hsa-mir-183 |
| ADIPOQ-AS1 | hsa-mir-182, hsa-mir-183, hsa-mir-184, hsa-mir-122, hsa-mir-375 |
| SACS-AS1 | hsa-mir-187 |
| EMX2OS | hsa-mir-182, hsa-mir-183, hsa-mir-184, hsa-mir-210 |
| BOK-AS1 | hsa-mir-184 |
| CHL1-AS1 | hsa-mir-137, hsa-mir-187 |
| LINC00484 | hsa-mir-141, hsa-mir-200a, hsa-mir-187, hsa-mir-122 |
| ARHGEF7-AS2 | hsa-mir-187, hsa-mir-210, hsa-mir-122, hsa-mir-375 |
| RBMS3-AS3 | hsa-mir-96, hsa-mir-182 |
| LINC00445 | hsa-mir-375 |
| PDZRN3-AS1 | hsa-mir-141, hsa-mir-200a |
| AL109754.1 | hsa-mir-122 |
| MME-AS1 | hsa-mir-182, hsa-mir-429 |
| ADAMTS9-AS1 | hsa-mir-301b, hsa-mir-96, hsa-mir-182, hsa-mir-21 |
| ADAMTS9-AS2 | hsa-mir-301b, hsa-mir-96, hsa-mir-137, hsa-mir-141, hsa-mir-200a, hsa-mir-182, hsa-mir-183, hsa-mir-184, hsa-mir-122, hsa-mir-375 |
| ALDH1L1-AS2 | hsa-mir-301b, hsa-mir-210 |
| AC040173.1 | hsa-mir-96, hsa-mir-182, hsa-mir-183, hsa-mir-429 |
| PWRN1 | hsa-mir-137, hsa-mir-184, hsa-mir-21, hsa-mir-122 |
| C2orf48 | hsa-mir-204 |
| SHANK2-AS3 | hsa-mir-145, hsa-mir-204 |
| C15orf54 | hsa-mir-206 |
| AC127496.1 | hsa-mir-204 |
| MIR7-3HG | hsa-mir-145, hsa-mir-204 |
| LINC00305 | hsa-mir-144, hsa-mir-204 |
| C10orf91 | hsa-mir-204 |
| WT1-AS | hsa-mir-145, hsa-mir-206 |
| LINC00518 | hsa-mir-145, hsa-mir-206, hsa-mir-204 |
| LINC00221 | hsa-mir-204 |
| TCL6 | hsa-mir-144, hsa-mir-145, hsa-mir-206, hsa-mir-204 |
| AF241725.1 | hsa-mir-145 |
| MUC2 | hsa-mir-145 |
| RMRP | hsa-mir-206 |
| C1orf137 | hsa-mir-204 |
| AL391421.1 | hsa-mir-144 |
| MUC19 | hsa-mir-144, hsa-mir-145, hsa-mir-206, hsa-mir-204 |
| UCA1 | hsa-mir-206 |
| LINC00488 | hsa-mir-144, hsa-mir-206 |
| LINC00243 | hsa-mir-145, hsa-mir-206 |
| AL356310.1 | hsa-mir-206 |
| SMCR2 | hsa-mir-145, hsa-mir-204 |
| LINC00466 | hsa-mir-144, hsa-mir-206, hsa-mir-204 |
| LINC00337 | hsa-mir-145 |
| LINC00113 | hsa-mir-145 |
| NAALADL2-AS2 | hsa-mir-206 |
| HOTAIR | hsa-mir-206, hsa-mir-204 |
| SRGAP3-AS2 | hsa-mir-145, hsa-mir-206 |
| LINC00200 | hsa-mir-204 |
| ATXN8OS | hsa-mir-145, hsa-mir-204 |
| DLX6-AS1 | hsa-mir-144, hsa-mir-145, hsa-mir-206, hsa-mir-204 |
| LINC00210 | hsa-mir-206, hsa-mir-204 |
| TLR8-AS1 | hsa-mir-206, hsa-mir-204 |
| LINC00460 | hsa-mir-206 |
| FNDC1-IT1 | hsa-mir-144 |
| DSCAM-AS1 | hsa-mir-204 |
| NDP-AS1 | hsa-mir-145, hsa-mir-206 |
| CLRN1-AS1 | hsa-mir-206, hsa-mir-204 |
| AC061992.1 | hsa-mir-204 |
| LINC00461 | hsa-mir-144, hsa-mir-145, hsa-mir-204 |
| MAST4-IT1 | hsa-mir-204 |
| LINC00536 | hsa-mir-204 |
| LINC00491 | hsa-mir-145, hsa-mir-204 |
| AL589642.1 | hsa-mir-145, hsa-mir-204 |
| LINC00524 | hsa-mir-204 |
| LINC00052 | hsa-mir-145 |
| LINC00261 | hsa-mir-144, hsa-mir-145, hsa-mir-206, hsa-mir-204 |

**Table S2 miRNAs targeting mRNAs of BC**

| **mRNA** | **mRNA** |
| --- | --- |
| hsa-mir-137 | KIT |
| hsa-mir-144 | KPNA2 |
| hsa-mir-182 | CHL1, TCEAL7 |
| hsa-mir-183 | AKAP12, SH3D19 |
| hsa-mir-204 | CDH2 |
| hsa-mir-21 | SPRY2 |
| hsa-mir-210 | SERTM1 |
| hsa-mir-429 | WASF3 |

**Table S3 Prognostic value of the lncRNAs analyzed by univariate Cox's proportional hazards regression model**

| **lncRNA** | **HR(95% CI)** | ***P* Value** |
| --- | --- | --- |
| ADAMTS9-AS1 | 0.84(0.74-0.96) | 0.008 |
| AC061992.1 | 0.85(0.74-0.98) | 0.030 |
| LINC00536 | 1.15(1.05-1.27) | 0.004 |
| HOTAIR | 1.07(1-1.15) | 0.037 |
| AL391421.1 | 0.92(0.86-0.99) | 0.034 |
| TLR8-AS1 | 1.14(1.01-1.3) | 0.041 |
| LINC00491 | 1.15(1.03-1.28) | 0.014 |
