# Supplementary figures and images for: Systematic analysis of lncRNA–miRNA–mRNA competing endogenous RNA network identifies four-lncRNA signature as a prognostic biomarker for breast cancer
Source: J Transl Med. 2018 Sep 27;16:264. doi: 10.1186/s12967-018-1640-2 (PMC6161429; doi:10.1186/s12967-018-1640-2)

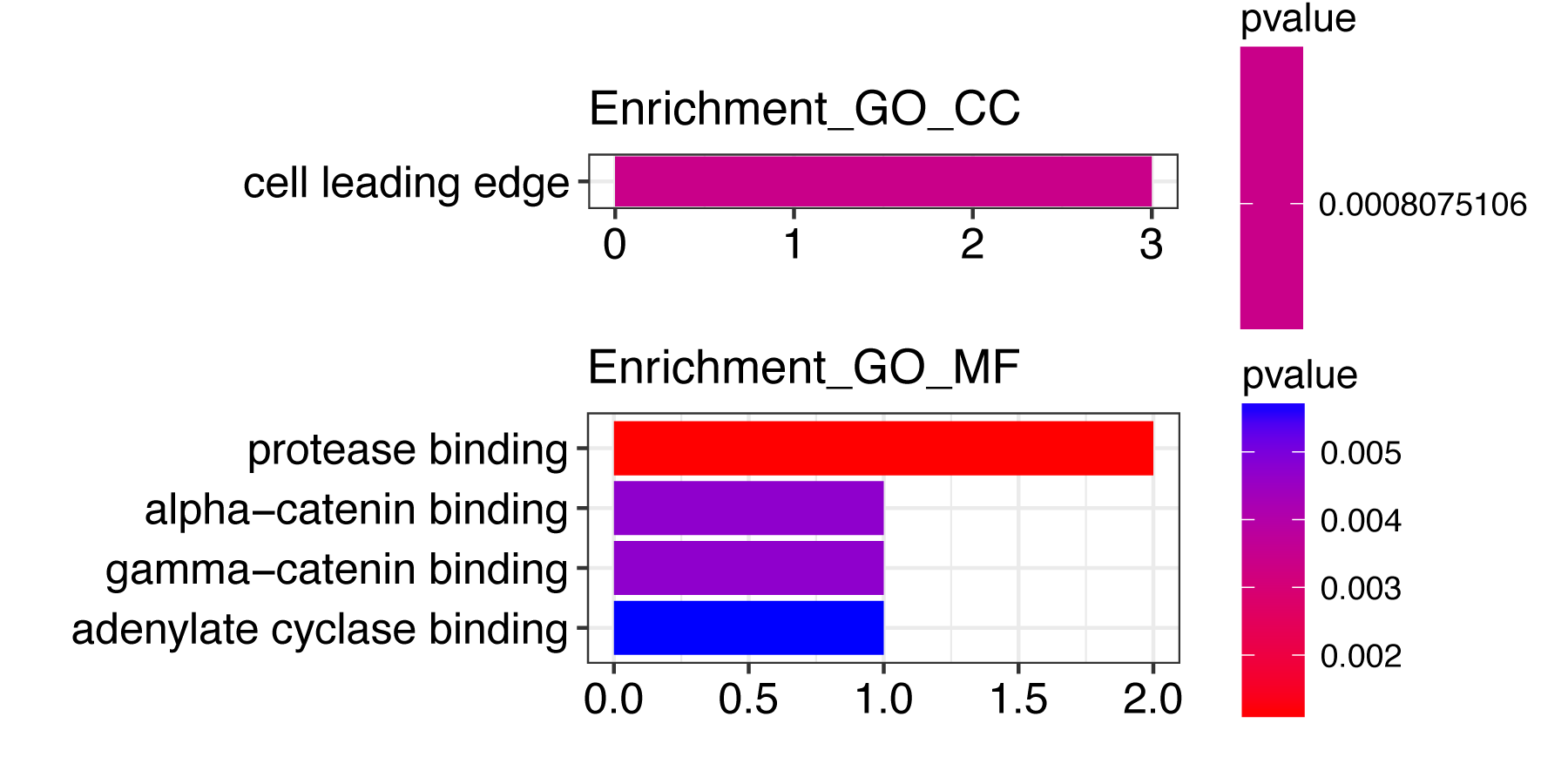

Supplement: Supplementary file 2 — Additional file 2: Figure S1. Gene Ontology (GO) analysis. GO results for aberrantly expressed mRNAs with significant Enrichment score covering domains of cellular components (CCs) and molecular functions (MFs). The bar plot devotes the enrichment scores of the significant GO terms. [file 12967_2018_1640_MOESM2_ESM.tif]

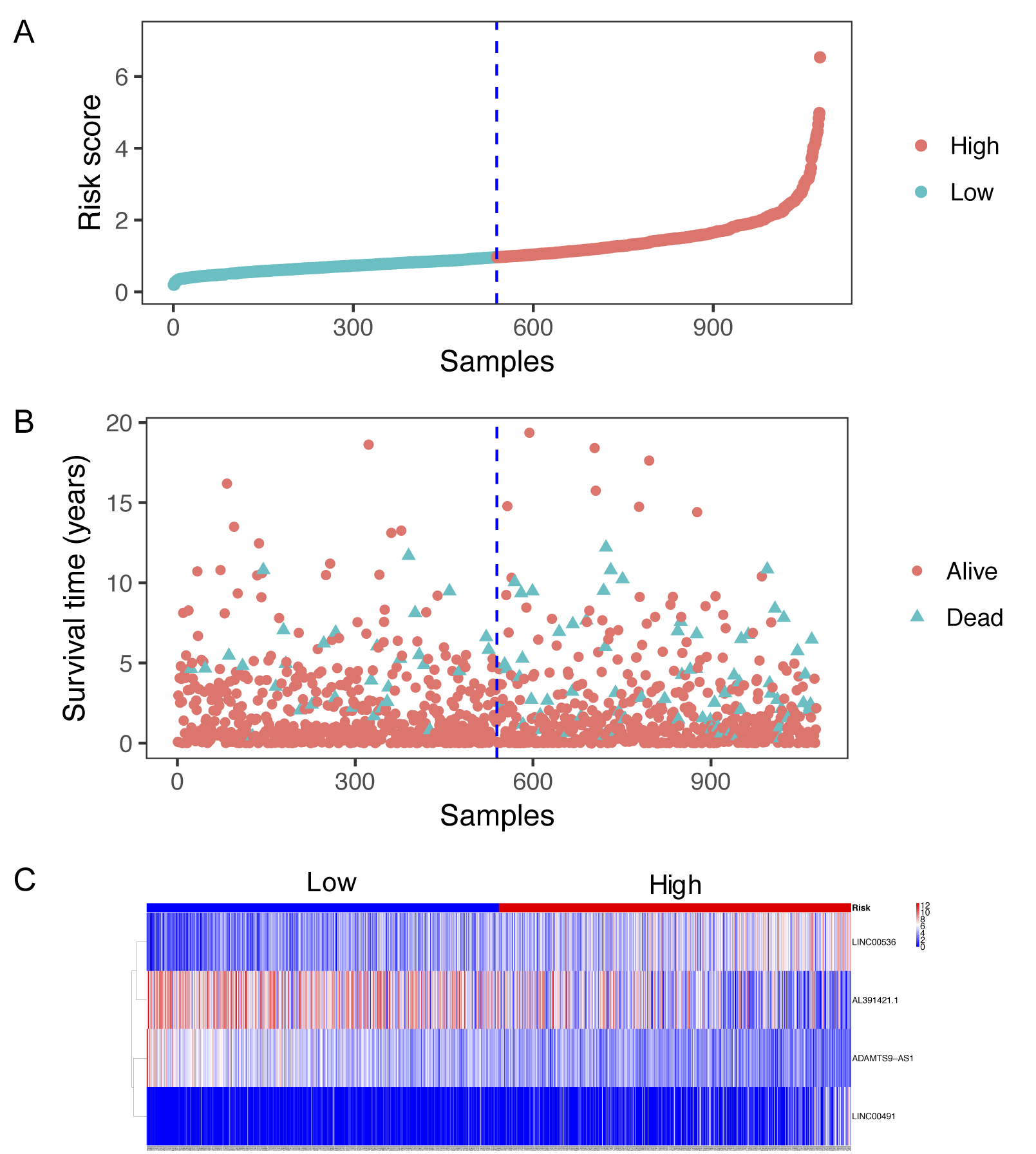

Supplement: Supplementary file 3 — Additional file 3: Figure S2. Four-lncRNA signature ((ADAMTS9-AS1, AL391421.1, LINC00491, and LINC00536)) predicted OS in BC cohort. A. Risk-score distribution. Red demonstrating higher expression while blue representing lower expression. Risk scores for all BC patients were created in ascending order and blue is marked as low risk or red is labeled as high risk. B. Patients’ survival status with blue devoting dead, and red standing for alive. C. Heat map of the four-lncRNA expression profiles in BC patients. [file 12967_2018_1640_MOESM3_ESM.tif]
